# Dataset on hydration, microstructure, mechanical performance and environmental impacts of Portland cement incorporating waste-derived ferrous sulfate

**DOI:** 10.1016/j.dib.2026.112863

**Published:** 2026-05-22

**Authors:** Bilguun Mend, Youngjun Lee, Jeong-Hwan Bang, Jang-Ho Jay Kim, Yong-Sik Chu

**Affiliations:** a Climate and Energy R&D Group, Korea Institute of Ceramic Engineering and Technology, Jinju 52581, Republic of Korea; b School of Civil and Environmental Engineering, Yonsei University, Seoul 03722, Republic of Korea

**Keywords:** Ferrous sulfate, Cement dataset, Gypsum replacement, Hydration kinetics, Mechanical performance, Chromium leaching, Cradle-to-gate assessment

## Abstract

This data article presents experimental datasets generated from ordinary Portland cement systems incorporating waste-derived ferrous sulfate hydrates (FeSO₄·xH₂O) as an alternative sulfate-regulating component to natural gypsum. The datasets document material synthesis conditions, cement binder preparation, hydration-related characteristics, microstructural features, mechanical performance, chromium speciation, and environmental inventory inputs associated with the investigated cement systems. The dataset includes X-ray diffraction (XRD) patterns and quantitative phase analysis files, thermogravimetric and derivative thermogravimetric (TG/DTG) data obtained over a broad temperature range, and scanning electron microscopy (SEM) images of hydrated cement pastes. Chemical composition data for raw materials and cement binders were obtained using X-ray fluorescence (XRF) and inductively coupled plasma optical emission spectroscopy (ICP-OES). Additional datasets include Vicat initial and final setting-time results, compressive strength measurements obtained at different curing ages, soluble hexavalent chromium [Cr(VI)] concentration data, and life-cycle inventory inputs compiled for the cradle-to-gate environmental assessment. All data files are systematically organized according to experimental technique, sample designation, and curing age, and include both raw instrument outputs and processed data tables to support transparency, reproducibility, and reuse. These datasets are intended to facilitate data-driven analysis, comparative evaluation of alternative sulfate-regulating additives, modelling of cement hydration behavior, and environmental assessment of cementitious materials incorporating industrial by-products.

Specifications Table

**Table:** 

Subject	Engineering & Materials science
Specific subject area	Cement hydration, alternative sulfate regulation, chromium speciation, and waste-derived cement additives.
Type of data	Tables, images, charts, graphs, figures, raw instrumental outputs, processed datasets, and analysed data.
Data collection	Data were collected from laboratory-scale cement binders incorporating waste-derived ferrous sulfate hydrates. XRD, TG/DTG, SEM, XRF, and ICP-OES analyses were conducted to characterize hydration, microstructure, chemical composition, and chromium-related data. Initial and final setting times and compressive strength were determined according to ASTM C191 and ASTM C109/C109M, respectively.
Data source location	Data were generated from laboratory-scale cement binders incorporating waste-derived ferrous sulfate hydrates. Material synthesis, cement binder preparation, physical and mechanical testing, microstructural and chemical analyses, and life-cycle inventory data compilation were conducted at the Climate and Energy R&D Group, Korea Institute of Ceramic Engineering and Technology (KICET), Jinju, Republic of Korea, and the School of Civil and Environmental Engineering, Yonsei University, Seoul, Republic of Korea.
Data accessibility	Repository name: Mendeley Data Data identification number: doi: 10.17632/7y6nzphndz.2 Direct URL to data: https://data.mendeley.com/datasets/7y6nzphndz/1
Related research article	Mend, B., Lee, Y., Bang, J.-H., Kim, J.-H. J., Chu, Y.-S. Performance and Environmental Assessment of Portland Cement Incorporating Waste-Derived Ferrous Sulfate as a Gypsum Substitute: A Case Study. Case Studies in Construction Materials, https://doi.org/10.1016/j.cscm.2026.e05909

## 1 Value of the Data

• These data provide detailed experimental records describing hydration-related measurements, phase assemblages, microstructural features, mechanical testing outputs, chromium speciation, and environmental inventory inputs for Portland cement systems incorporating waste-derived ferrous sulfate hydrates as sulfate-regulating components.
• These datasets may benefit cement manufacturers, ready-mix concrete producers, construction-materials testing laboratories, environmental consultants, regulatory agencies concerned with soluble Cr(VI) in cement-based materials, and researchers working on cement chemistry, waste valorization, and sustainable construction materials.
• The datasets enable reuse for comparative analysis of alternative sulfate sources in cementitious materials, including benchmarking against conventional gypsum-regulated systems under similar curing and testing conditions.
• Raw XRD, TG/DTG, SEM, chemical composition, and mechanical datasets can be reanalyzed using different analytical, statistical, or modeling approaches, supporting validation and development of hydration, phase-equilibrium, and microstructure-based models.
• Chromium speciation data support reuse in studies examining redox-active additives, cement chemistry related to trace elements, and regulatory compliance assessments associated with soluble Cr(VI) in cement-based materials.
• Life-cycle inventory datasets provide reusable inputs for environmental assessment, scenario analysis, and sustainability studies focused on waste-derived cement components and alternative material sourcing strategies.

## 2 Background

The dataset was compiled to document experimental measurements associated with the use of waste-derived ferrous sulfate hydrates (FeSO₄·xH₂O) as an alternative sulfate source in ordinary Portland cement systems [1]. Sulfate regulation plays a central role in controlling early-age hydration processes, phase assemblage development, and setting behavior of cementitious materials [2]. Conventional sulfate regulation relies primarily on natural gypsum, motivating the exploration of alternative sulfate-bearing materials derived from industrial by-products [3]. Ferrous sulfate hydrates can be generated from iron-rich industrial wastes through controlled chemical processing, producing materials with distinct chemical composition and hydration-related behavior compared to conventional gypsum [4]. Generating structured datasets describing hydration-related measurements, microstructural observations, mechanical testing outputs, chromium-related chemical and speciation data, and environmental inventory inputs provides a foundation for transparent reporting and reproducibility of cement-related experiments involving alternative sulfate sources [5,6]. This data article was prepared to organize and make accessible the raw and processed outputs obtained from standardized laboratory procedures, including diffraction, thermal analysis, microscopy, mechanical testing, chemical analysis, chromium-related instrumental analysis, and life-cycle inventory compilation. The dataset enables independent reanalysis, methodological comparison, and reuse in data-driven investigations of cement systems incorporating waste-derived sulfate-bearing components. Such datasets are relevant to cement manufacturers, construction-material researchers, environmental assessment practitioners, and regulatory stakeholders interested in alternative sulfate regulation, chromium control, and waste-derived cement additives.

## 3 Data Description

The dataset is organized into folders according to experimental technique and data type. Each folder contains raw instrument outputs, processed files, and accompanying metadata, allowing users to follow the structure and content of the dataset without reference to additional sources. An overview of the repository structure and the main data files is provided in Table 1.

**Table 1:** Overview of dataset folders and data files provided in the repository.

Folder name	Data type	Main contents	Output provided
S1_XRD	Raw and processed data	X-ray diffraction patterns and quantitative phase analysis	Raw instrument files and processed phase tables
S2_TG	Raw and processed data	TG/DTG curves recorded from 40 to 1050°C	Mass-loss curves and derivative data tables
S3_SEM	Image data	SEM images of hydrated cement pastes with visible scale bars	Image files at different magnifications
S4_XRF	Chemical composition data	XRF raw output files for LD sludge, OPC clinker, and FeSO₄·xH₂O residues where applicable	Raw XRF output files and chemical composition data
S5_Compressive_Strength	Mechanical data	Compressive strength at 3, 7, 14, and 28 days	Individual specimen values and calculated averages
S6_Setting_time	Test data	Vicat initial and final setting-time results	Processed setting-time tables
S7_Cr_VI_Reduction	Chromium speciation data	Soluble Cr(VI) concentration data	Raw measurement outputs and processed concentration tables
S8_LCI	Environmental inventory data	Cradle-to-gate material, energy, and process inputs	Life-cycle inventory tables

### 3.1 Hydration, microstructural, and mechanical datasets

The hydration, microstructural, and mechanical datasets are provided in the S1_XRD, S2_TG, S3_SEM, S4_XRF, S5_Compressive_strength, and S6_Setting_time folders. These folders contain raw and processed data related to phase evolution, thermal decomposition behavior, microstructural features, chemical composition, mechanical performance, and setting behavior of the investigated cement systems.

**Fig. 1:**
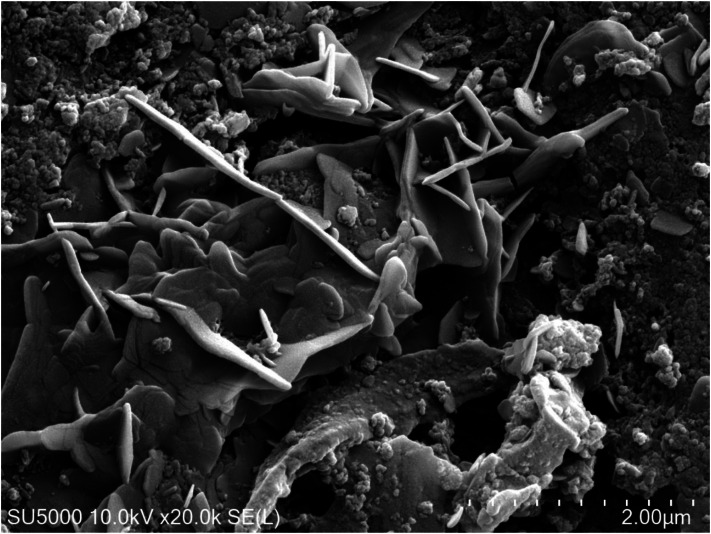
Representative SEM image of hydrated cement paste incorporating waste-derived ferrous sulfate hydrate. The scale bar is clearly indicated as 2.00 µm.
• The S4_XRF folder provides XRF raw output files for LD sludge, OPC clinker, and FeSO₄·xH₂O residues where applicable.
• The S5_Compressive_strength folder contains raw and averaged compressive strength measurements for Control, F25, F50, F75, and F100 samples at 3, 7, 14, and 28 days.
• The S6_Setting_time folder includes initial and final setting-time measurements, replicate data, and raw Vicat penetration logs where available.

• The S1_XRD folder contains raw X-ray diffraction scans of hydrated cement pastes, FeSO₄·xH₂O synthesis products, LD sludge, and residue samples. Where available, Rietveld refinement outputs are also provided to support phase identification and quantitative phase analysis.
• The S2_TG folder includes TG and DTG raw datasets, together with processed mass-loss tables for hydration-related phases such as AFt/AFm, CH, and CaCO₃.
• The S3_SEM folder contains high-resolution SEM images of Control and F50 cement pastes. The image files were revised to include clearly visible scale bars and scale labels for reliable microstructural interpretation. A representative SEM image is shown in Fig. 1, while the complete SEM image set is provided in the repository.

### 3.2 Chromium-related and environmental datasets

The chromium-related and environmental datasets are provided in the S7_Cr_VI_reduction and S8_LCI folders, respectively. These datasets are summarized in Table 2 to clarify the location, analytical basis, main contents, and outputs of the Cr(VI)/ICP-OES and life-cycle inventory data. The S7_Cr_VI_reduction folder contains ICP-OES results for soluble hexavalent chromium [Cr(VI)] after alkaline extraction, FeSO₄·xH₂O dosage-dependent Cr(VI) reduction data, and comparison data related to the EU 2 ppm Cr(VI) limit. These files provide traceable chromium-related data for evaluating the effectiveness of waste-derived ferrous sulfate hydrates in reducing soluble Cr(VI) in cement-based systems. The S8_LCI folder provides life-cycle inventory data for waste-derived FeSO₄·xH₂O production, including waste H₂SO₄, LD sludge, process water, energy, electricity, transport inputs, and impact indicators for comparison with the natural gypsum baseline. These datasets support reuse in cradle-to-gate environmental assessment and scenario analysis of waste-derived sulfate-regulating cement additives.

**Table 2:** Summary of chromium-related and environmental datasets provided in the repository.

Dataset folder	Dataset category	Method / basis	Main contents	Output provided
S7_Cr_VI_reduction	Chromium-related dataset	Alkaline extraction and ICP-OES analysis	Soluble hexavalent chromium [Cr(VI)] concentration data, FeSO₄·xH₂O dosage-dependent Cr(VI) reduction data, and comparison with the EU 2 ppm Cr(VI) limit	Raw ICP-OES results, processed concentration tables, and regulatory comparison data
S8_LCI	Environmental inventory dataset	Cradle-to-gate life-cycle inventory compilation	Inventory data for waste-derived FeSO₄·xH₂O production, including waste H₂SO₄, LD sludge, process water, energy, electricity, transport inputs, and impact indicators for comparison with natural gypsum	Life-cycle inventory tables and processed environmental assessment inputs

## 4 Experimental Design, Materials and Methods

### 4.1 Material synthesis and cement binder preparation

The datasets were generated through a controlled laboratory workflow designed to ensure reproducibility and traceability of data acquisition. Waste-derived ferrous sulfate hydrates (FeSO₄·xH₂O) were produced from LD steelmaking sludge and waste sulfuric acid using a cyclic reaction–filtration process. The process involved acidic dissolution under elevated-temperature conditions, solid–liquid separation, controlled cooling to induce crystallization, and low-temperature drying. Synthesis parameters were recorded during data acquisition to support replication of material preparation. Cement binders were prepared by replacing natural gypsum with predefined proportions of waste-derived FeSO₄·xH₂O. Raw materials were homogenized and ground under controlled milling conditions to obtain comparable fineness before sample preparation. Cement pastes and mortars were prepared using fixed mixture proportions and cured under standardized temperature and humidity conditions. Sample identification codes were assigned according to mixture composition and curing age to ensure consistency across the datasets.

### 4.2 Hydration, microstructural, and chemical analyses

Phase-related datasets were acquired using X-ray diffraction under standardized scan conditions, and thermal datasets were obtained using thermogravimetric and derivative thermogravimetric analysis under controlled heating conditions. Microstructural datasets were generated from fractured cement paste samples after hydration arrest and drying. SEM images were acquired at selected curing ages and magnifications, and revised image files include clearly visible scale bars and scale labels to support reliable microstructural interpretation. Chemical composition datasets were generated using X-ray fluorescence (XRF) and inductively coupled plasma optical emission spectroscopy (ICP-OES). Chromium-related datasets were obtained using ICP-OES after alkaline extraction of soluble hexavalent chromium [Cr(VI)]. Raw ICP-OES outputs and processed concentration tables were retained in the repository to support traceability, verification, and reuse of the chromium-related data.

### 4.3 Mechanical, setting-time, and environmental inventory datasets

Mechanical and setting-related datasets were obtained using standardized test configurations, specimen geometries, and curing durations. Initial and final setting times were measured using the Vicat method, and compressive strength was determined at selected curing ages using mortar specimens. Individual measurements and calculated average values were retained in the corresponding repository folders. Environmental inventory data were compiled by recording material inputs, energy consumption, transport-related inputs, and process parameters associated with waste-derived FeSO₄·xH₂O production and cement binder preparation. These inventory data were organized for cradle-to-gate environmental assessment and comparison with the natural gypsum baseline.

All raw instrument outputs were retained in their original formats. Processed data files were generated using consistent calculation procedures, and no filtering or exclusion criteria were applied beyond standard instrument quality controls. The complete dataset was organized by supplementary folder, experimental technique, sample designation, and acquisition sequence to support transparent reuse of the data.

## Limitations

The datasets were generated under controlled laboratory-scale conditions using specific raw materials and processing parameters. Therefore, variability associated with industrial-scale production, different clinker compositions, alternative waste sources, or large-scale processing conditions is not represented. Hydration, microstructural, mechanical, setting-time, and chromium-related datasets were collected for selected mixture proportions and curing ages. Thus, the data may not fully capture long-term performance, continuous hydration development, or behavior under different curing regimes. The Cr(VI) and ICP-OES datasets are limited to the extraction procedures, analytical conditions, and sample compositions used in this study. SEM images provide representative microstructural observations at selected magnifications, and the life-cycle inventory data reflect laboratory-scale material synthesis and processing records. No data filtering or exclusion was applied beyond standard instrument quality controls. Users should consider measurement uncertainty and scale-related limitations when reusing the data for alternative materials, environmental assessment, or regulatory comparison.

## Ethics Statement

This work did not involve human participants, animal experiments, or the use of data collected from social media platforms. Therefore, no ethical approval or informed consent was required.

## CRediT authorship contribution statement

**Bilguun Mend:** Conceptualization, Methodology, Writing – original draft, Writing – review & editing. **Youngjun Lee:** Writing – original draft, Visualization. **Jeong-Hwan Bang:** Writing – original draft, Validation. **Jang-Ho Jay Kim:** Supervision, Validation. **Yong-Sik Chu:** Writing – review & editing, Supervision, Validation.

## Data Availability

(Mendeley Data).Dataset on hydration, microstructure, mechanical performance and environmental impacts of Portland cement incorporating waste-derived ferrous sulfate (Original data)
